# Acoustic Roughness Measurement of Railway Tracks: Running Surface Detection and Compensation of Lateral Movements for Optical Measurements on a Train

**DOI:** 10.3390/s23125764

**Published:** 2023-06-20

**Authors:** Florian Mauz, Remo Wigger, Loris Griesbaum, Tobias Wahl, Michal Kuffa, Konrad Wegener

**Affiliations:** 1 Institute for Machine Tools and Manufacturing, ETH Zürich, 8092 Zurich, Switzerland; 2 Inspire AG, 8005 Zurich, Switzerland

**Keywords:** railway rolling noise, rail profiles, acoustic roughness, condition monitoring, chord method, laser triangulation, laser profilometer

## Abstract

Rolling noise is a significant contributor to railway noise. Wheel and rail roughness are decisive for the emitted noise level. An optical measurement method installed on a moving train is suitable for closer monitoring of the rail surface condition. A measurement setup based on the chord method requires the sensors to be positioned in a straight line along the direction of measurement and in a stable lateral position. Measurements should always be performed within the shiny and uncorroded running surface, even when there are lateral movements of the train. In this study, concepts for the detection of the running surface and the compensation of lateral movements are investigated in a laboratory setting. The setup consists of a vertical lathe with a ring-shaped workpiece that incorporates an implemented artificial running surface. The detection of the running surface based on laser triangulation sensors and a laser profilometer is investigated. It is shown that the running surface can be detected using a laser profilometer that measures the intensity of the reflected laser light. It is possible to detect the lateral position and the width of the running surface. A linear positioning system is proposed to adjust the lateral position of the sensors based on the running surface detection of the laser profilometer. When the lateral position of the measuring sensor is disturbed by a movement with a wavelength of 18.85 m, the linear positioning system can keep the laser triangulation sensor inside the running surface for 98.44% of the measured data points at a velocity of approximately $7.5\;{\mathrm{km}\;h}^{-1}$. The mean positioning error is 1.40 mm. By implementing the proposed system on the train, future studies can be conducted to examine the lateral position of the running surface as a function of the various operational parameters of the train.

## 1. Introduction

**Figure 1:**
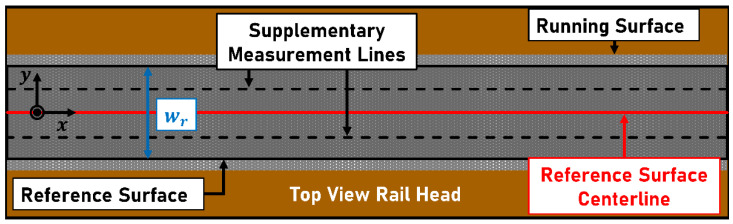
Top view of a rail head with measurement lines within the running surface as defined in EN 15610 [19]. $w_{r}$: width of the reference surface.

Railway noise can harm human health. Pyko et al. [1] and Vienneau et al. [2] found a correlation between long-term noise exposure and increased incidence of cardiovascular diseases. Rolling noise is dominant in the velocity range between $60\;{\mathrm{km}\;h}^{-1}$ and $300\;{\mathrm{km}\;h}^{-1}$, as described by Müller et al. [3]. Thompson [4] determined the correlation between the roughness of the wheel, the rail, and the generated rolling noise. Rolling noise can be reduced by acoustic rail grinding, as described by Kuffa et al. [5]. Grassie [6] expressed the need for measurements to monitor the quality of the grinding process. The condition of the network must be monitored to achieve optimal rail maintenance. Numerous methods have been developed to measure the longitudinal rail profile and the acoustic roughness of rails. Cordier et al. [7] distinguished between direct and indirect measuring methods. Direct methods measure the longitudinal rail profile, e.g., by using a tactile measurement. Grassie et al. [8] developed the Corrugation Analysis Trolley (CAT) device to measure rail irregularities. Tanaka et al. [9] developed a hand-pushed trolley to perform direct measurements of the rail roughness using laser distance sensors based on the chord method. Instead of using laser distance sensors, a laser profilometer can be applied in the longitudinal direction as described by Teng et al. [10]. Laser profilometers were applied in the transverse direction by Boronahin et al. [11] to measure the rail profile. An automated trolley was presented by Jeong et al. [12]. Indirect measuring methods measure parameters that are indicative of the rail roughness. Hauck et al. [13] described the measurement of rolling noise to determine the rail condition concerning acoustic emissions. Kuijpers et al. [14] determined rail roughness levels from measured rolling noise and mentioned the need for calibration, which depends on track properties. Alternatively, axle-bearing accelerations are measured to determine the roughness levels of the rail. Bongini et al. [15] calibrated the High-Speed Rail Corrugation Analyzer (HSRCA) system using a CAT trolley and mentioned the dependence of the system on the measuring speed as well as on the dynamic properties of the track. Carrigan et al. [16] presented a method to remove the influences of wheel roughness on the measurement result. Unlike indirect measuring methods, direct measurements cannot be performed at line speed with currently available measurement devices. The advantages of direct and indirect measurements could be combined by performing optical measurements of the rail roughness from a moving train. Mauz et al. [17] evaluated an optical approach based on the chord method to measure rail roughness directly under laboratory conditions. Vertical disturbances of the train and environmental influences were simulated. Lateral movements of the train were not considered, and the measurements were performed on one fixed lateral position above the rail. Mauz et al. [18] applied the measurement concept on a test train. The measurement system was mounted to the train bogie. A fixed lateral measuring position could not be maintained due to lateral movements of the train, such as hunting motion or the passing of switches. In addition to the lateral movements of the rail vehicle, a displacement of the running surface (shiny and uncorroded part of the rail), which transverses to the direction of travel along the track, can be observed. The chord method is based on the measurement on a fixed line and is preferably performed on the rail head centerline or at least within the running surface, since this is the area where the rolling contact occurs. EN 15610 [19] defines the number and position of the measuring lines for the calculation of the acoustic roughness and a direct measurement method. The width of the running surface and its position on the rail head surface can vary along the rail. The rail surface, divided into laterally shifted measuring lines, is shown in Figure 1.

The reference surface corresponds to the area of minimum width within the running surface over the selected measuring length. Depending on its width $w_{r}$, EN 15610 [19] defines the following lateral measuring positions:

- $w_{r}\leq 20\;\mathrm{mm}$*:* The longitudinal profile is measured in the center of the reference surface (on the centerline).
- $20\;\mathrm{mm}<w_{r}\leq 30\;\mathrm{mm}$: The longitudinal profile is measured on three lines. Two additional lines are at a 5 mm lateral distance to the centerline.
- $w_{r}>30\;\mathrm{mm}$: The longitudinal profile is measured on three lines. The additional measurement lines are placed at a 10 mm lateral distance to the centerline.

Measuring lines should be kept within a tolerance of ±1 mm.

Chen et al. [20] measured the longitudinal rail profile on the centerline and compensated for lateral movements by detecting the laser points of the measurement line with a camera system. The system could be operated up to a speed of $6\;{\mathrm{km}\;h}^{-1}$ with a maximum positioning error of 0.6 mm. Chen et al. [21] further developed the concept and used two laser lines to transverse the driving direction for the optical support of the camera due to illumination conditions. The positioning error in this application was 0.4 mm. Chen et al. [21] established a maximum driving speed of $9\;{\mathrm{km}\;h}^{-1}$.

The objective of this work is to detect the running surface and compensate for lateral train deflections using optical sensors. A laser profilometer and four laser triangulation distance sensors are available to detect the running surface. The following topics are addressed:

- Concepts for the compensation, respectively addressing the lateral movements of the optical rail roughness measurement system, are presented;
- Whether the stated sensor types are suitable for the detection of differences in surface conditions and the running surface is examined. The case of a rail with a running surface and corroded edges is assumed for all investigations;
- Lateral deflection is artificially added, and a lateral compensation system is tested on a test bench under laboratory conditions.

## 2. Materials and Methods

### 2.1. Concepts

Different concepts for the detection of the running surface and compensation of the lateral movements of the train are presented. The available selection of sensors (laser triangulation and laser profilometer) offers several concepts for the compensation, respectively addressing the lateral movements of the optical rail roughness measurement system:

**(1) Fixed Installations:** Approaches that only include non-moving sensors on a fixed lateral measuring position have an advantage in that no actuators are required. Since no moving parts are needed, less complex designs of the device are possible.

**Figure 2:**
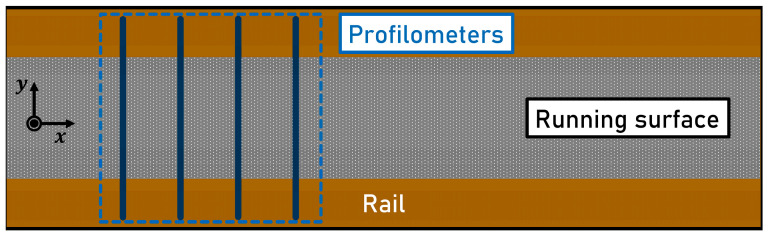
Top view of a rail head with four successive laser profilometers measuring the longitudinal profile as defined in concept 1 a.

- **Four Profilometers:** Instead of four laser triangulation sensors, four profilometers can be installed, which project lines onto the rail surface transverse to the direction of travel. The arrangement of the laser profilometers is shown in Figure 2.

**Figure 3:**
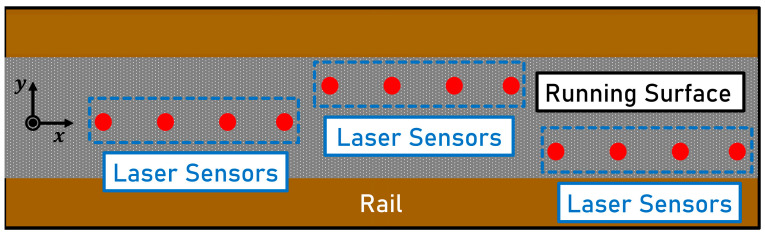
Top view of a rail head with three laterally shifted measurement lines, each consisting of four successive laser triangulation sensors measuring the longitudinal profile, as defined in concept 1 b.

In the case of a lateral offset of the train, a part of the line would always provide a measured sample within the running surface, assuming that the amplitude of the deflection is smaller than half the length of the projected line. The final longitudinal rail profile can be determined based on four sensor signals using the chord method. Additionally, the position and width of the running surface could be acquired if it is possible to determine these parameters from the measured data. When implementing this concept, the low sampling frequencies and the small measuring range with increasing sampling frequency are limiting factors. EN 15610 [19] requires a maximum longitudinal sampling distance of 1 mm. For a driving speed of $108{\;\mathrm{km}\;h}^{-1}$, a sampling frequency of 30 kHz would be required. The maximum sampling frequency of the available profilometer (Micro-Epsilon scanCONTROL 3060–50/BL) can reach 10 kHz if the measuring range is sufficiently reduced. This limits the maximum driving speed to $36{\;\mathrm{km}\;h}^{-1}$ before violating the requirement of EN 15610 [19]. Additionally, the maximum sampling frequency and the maximum driving speed are further reduced to 1294 Hz and $4.66\;{\mathrm{km}\;h}^{-1}$, respectively, if the measuring range is adjusted to 10 mm. This is the minimum necessary range for measurements of the longitudinal rail profile.

b. **Twelve Laser Triangulation Sensors** (**Three Measurement Lines**)**:** The measuring concept of Mauz et al. [18] measures the longitudinal profile on a single measuring line. Four consecutive laser triangulation sensors measuring the longitudinal profile at the identical lateral position are referred to as one measurement line. To stay within the running surface, the longitudinal profile can be measured on three lines, as shown in Figure 3, placed at different lateral positions instead of only one measuring line.

If the lateral distance between the lines is large enough, at least one line could be located within the running surface, assuming that the running surface width is sufficient. The measurement setup consisting of four laser triangulation sensors per measurement line would thus have to be extended to three lines and a total of twelve sensors. Since no cross-sectional profile can be recorded, consequently, it is not possible to determine the exact position of the running surface or its width.

**(2) Lateral Position Adjustment:** Compared to concept 1 b (Figure 3), the longitudinal profile is measured on one adjustable measuring line consisting of four consecutive sensors. Instead of twelve, only four laser triangulation sensors are required. The measurement setup is shifted laterally according to the position of the running surface. Additional moving parts and actuators are required. The clearance of linear guides and the oscillation behavior can lead to additional disturbances in the measurement result.

- **One Profilometer and Four Laser Triangulation Sensors:** A profilometer determines the position of the running surface. Four laser triangulation sensors measure the longitudinal profile on a measuring line which is positioned on the running surface using actuators.
- **Three Laser Triangulation Sensors and Four Laser Triangulation Sensors** (**One Measurement Line**)**:** Three laterally offset laser triangulation sensors indicate whether their line is on the running surface or not. Four laser triangulation sensors measure the longitudinal profile on a measuring line which is moved laterally using actuators. The signals of the three laterally offset sensors are used to determine the direction in which the laser sensors must be moved. This approach does not allow for the determination of the exact location of the running surface or its width since the surface state can only be detected in the discretized form at three lateral positions.

In the following, concept 2 a, that is, lateral position adjustment based on the detection of the running surface with a profilometer in combination with one measurement line of laser triangulation sensors, is investigated. The laser profilometer provides an opportunity to measure the width and lateral position of the running surface. The adjustable measuring line provides the possibility to set the lateral position on the centerline of the running surface.

### 2.2. Experimental Setup

**Figure 4:**
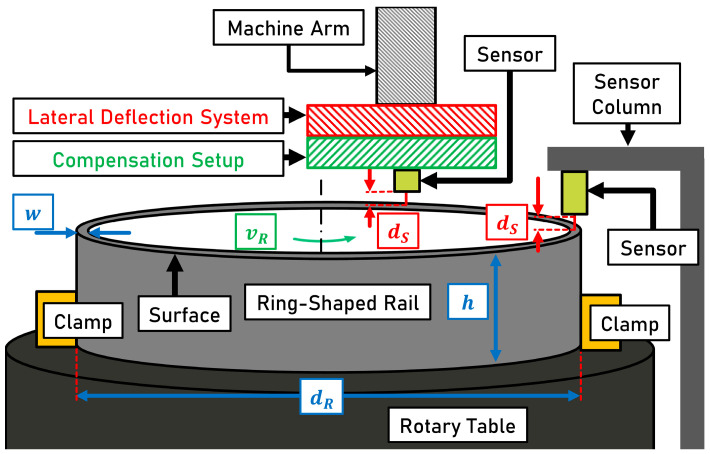
Schematic illustration of the testbench for validation of compensation approaches based on the setup described by Kuffa et al. [5]. w: width of the ring; h: height of the ring; $d_{R}$: average diameter of the ring; $v_{R}$: rotational speed of the ring; $d_{S}$: measurement distance of the respective sensor.

The test setup is shown schematically in Figure 4. A SEDIN 1525 vertical lathe serves as the basis for the experimental setup. The rotation of the ring-shaped workpiece can be used to simulate a train passing over a rail. The width w of the surface of the ring-shaped rail is 45 mm. The height h of the ring clamped on the rotary table is approximately 25 cm. The average diameter $d_{R}$ of the ring-shaped rail measures approx. 2 m. Measurements are conducted at a ring rotational speed $v_{R}$ of $20\;\min^{-1}$, which corresponds to a driving speed of $\sim 7.5\;{\mathrm{km}\;h}^{-1}$. Sensors can be mounted on the sensor column decoupled from the machine structure to minimize vibration. Surface condition detection tests (Section 2.3) are each performed with a single sensor (laser triangulation sensor or laser profilometer) mounted on the sensor column. All other tests are performed using attachments to the lateral deflection system. The machine arm of the vertical lathe provides the connection for the lateral deflection system. The lateral deflection system emulates the lateral displacements of the running surface relative to the train. The compensation setup is used to test different concepts related to the handling of lateral deflections.

**Figure 5:**
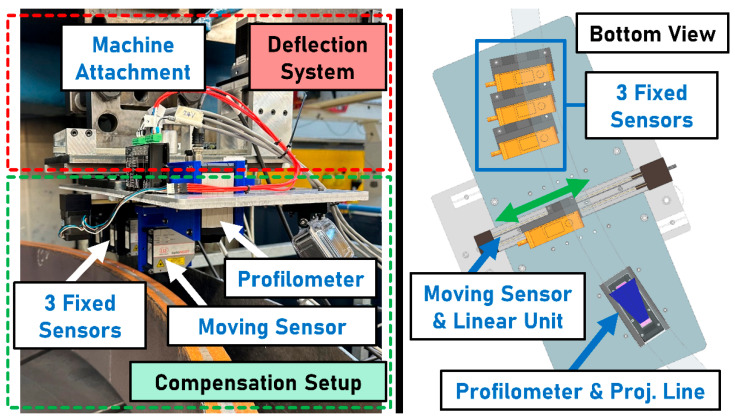
(**Left**): The experimental setup consists of the deflection system and the compensation setup. The setup includes one laser profilometer, one moving laser triangulation sensor, and three fixed laser triangulation sensors. (**Right**): Bottom view (CAD model) of the setup. When deflected, the moving sensor can adjust its lateral position to follow the running surface using a linear guide and a stepper motor.

The circumferential direction of the ring is labeled as longitudinal (x-axis). Movements and positions that transverse to the longitudinal direction are referred to as lateral (y-axis). The lateral deflection system and the compensation setup attached to it are shown in Figure 5.

The deflection system is actuated using a Microstep (MICROSTEP GmbH Schrittmotoren, Steuerungen, Bewegungssysteme, Sömmerda, Germany) GKS 56/200/9-4334 stepper motor. The applied amplitudes and frequencies of the lateral deflection are presented in Section 2.4. The lateral movement, actuated by a toothed belt, is guided by a system consisting of rails and slides. The moving sensor is actuated by an igus (igus GmbH, Cologne, Germany) ZLN-40 linear unit, including a stepper motor, to compensate for lateral deflection. The linear unit is controlled using a National Instruments (National Instruments Corporation, Austin, TX, USA) cRIO 9045 and an NI 9472 module. Measurements are performed with a Micro-Epsilon (Micro-Epsilon Messtechnik GmbH & Co. KG, Ortenburg, Germany) scanCONTROL 3060-50/BL laser profilometer (blue laser light) and a Micro-Epsilon optoNCDT 2300–10 LL laser triangulation sensor. The measuring distances and measuring ranges of the sensors are specified in Table 1.

The laser profilometer is mounted at a distance $d_{P}$ of 125 mm from the rail surface, which corresponds to the middle of its measuring range of 40 mm. The line consisting of 2048 measuring points projected in transverse direction has a length of 51 mm at the selected measuring distance. The profilometer has a linearity deviation of 3 μm. The distance and intensity of the light reflected to the receptor element are measured and transmitted via Ethernet. The intensity is measured as a value between 0 and 1023, representing minimum and maximum. A Micro-Epsilon optoNCDT 2300–10 LL laser triangulation sensor is used to measure the longitudinal profile or to detect the running surface. The sensor is mounted at a distance $d_{T}$ of 35 mm from the ring surface, which corresponds to the middle of its measuring range of 10 mm. The measurement resolution is 0.15 μm. The analog distance signal is recorded using an NI 9222 module while the digital intensity signal is transmitted via Ethernet. The intensity is measured as a value between 0% and 100%.

### 2.3. Surface Condition Detection

**Figure 6:**
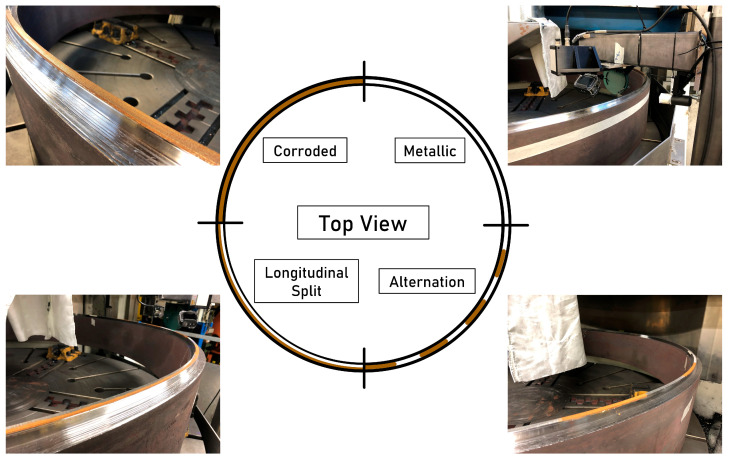
The ring surface applied for experimental analysis of surface condition detection. The ring is divided into four segments, each with a unique corrosion pattern. 1—metallic, 2—corroded, 3 —longitudinal split, 4—alternation.

To detect the smooth and shiny rail surface called the running surface using laser distance sensors, it is first necessary to determine the measurement variable relevant for the differentiation of surface conditions. A laser triangulation sensor offers two reasonable measurement variables for this purpose: the distance to the workpiece and the intensity of the light spot reflected to the receptor element. The respective sensors are mounted at measurement distances stated in Table 1. The setup is installed on the sensor column to exclude the influence of machine vibrations. The ground ring surface is artificially corroded as an imitation of a recently ground rail head with already corroded surface areas. Artificial corrosion is generated by applying a hydrogen peroxide-salt solution three times, accelerating the corrosion process. The partially corroded surface of the ring is shown in Figure 6.

The quarter segment with a metallic surface represents a measurement inside the running surface while the quarter segment with a corroded surface represents a measurement outside the running surface. The quarter segment in which the ring surface is divided longitudinally between the corroded and metallic areas represents a measurement in the edge area of the running surface. The sudden change between the running surface and the corroded area of the rail surface is represented by the alternation of corroded and metallic surface parts within the last quarter segment. The laser profilometer projects a line transverse to the direction of travel while the laser triangulation sensor measures on the centerline of the ring surface. The laser triangulation sensor measures distance and intensity with a sampling frequency $f_{S}$ of 20 kHz. With a ring speed of $20\;\min^{-1}$, the longitudinal data point distance $dx_{S}$ becomes 0.105 mm. The laser profilometer measures distance and intensity at a sampling frequency $f_{p}$ of 450 Hz and an exposure time of 0.4 ms. This results in a longitudinal data point distance $dx_{P}$ of 4.654 mm. Each experiment is repeated three times.

### 2.4. Running Surface Detection and Lateral Compensation Approach

**Figure 7:**
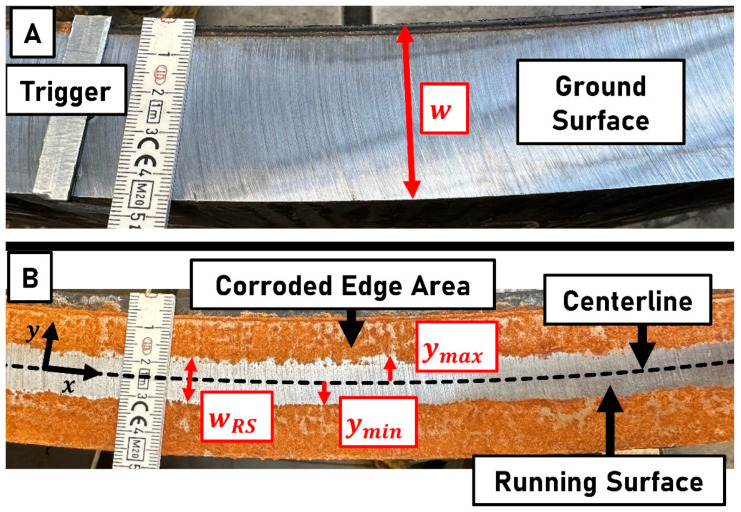
(**A**) The ground ring surface before application of artificially corroded edge areas. w: Width of the ring. (**B**) The ground ring surface after application of corroded edge areas as an imitation of a recently ground rail head with already corroded edge areas and therefore a visible running surface. $w_{RS}$: width of the running surface; $y_{min}$: left boundary of the running surface; $y_{max}$: right boundary of the running surface.

**Figure A1:**
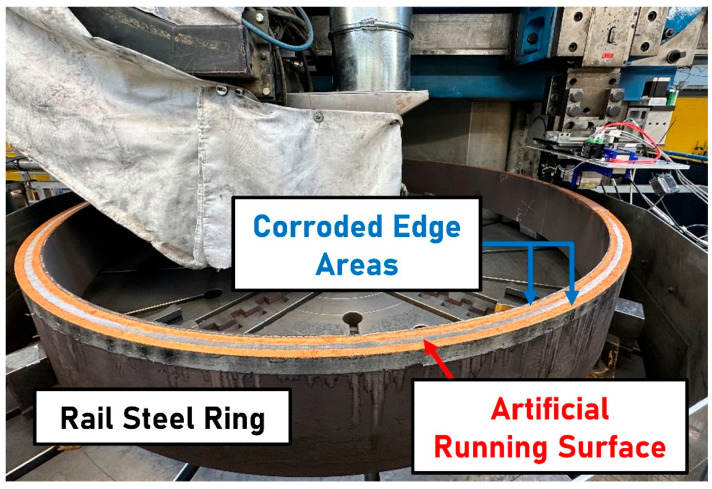
The mounted rail steel ring on the rotating table and the artificially created running surface.

The setup shown in Figure 5 is applied for the running surface detection and lateral compensation investigations. It consists of the lateral deflection system and the sensors attached to the machine arm. An artificial running surface is created on the ground and smooth ring surface. The surface condition is comparable to a rail that was ground a few days ago and already has corroded edge areas. The running surface does not include impurities, small accumulations of corrosion, or indentations. The setup including the entire ring and its prepared surface is shown in Figure A1. The tactile surface roughness of an imprint of the ring surface that is 100 mm in length is measured using a Taylor Hobson Talysurf. The roughness $R_{a}$ is determined for an evaluation length of 12.5 mm and with cutoff lengths $l_{s}$ of 8 μm and $l_{c}$ of 2.5 mm. The ground ring surface before and after application of the corroded edge areas is shown in Figure 7.

The width of the artificial running surface $w_{RS}$ measures approx. 10−12 mm. The laser profilometer measures the intensity with a sampling frequency $f_{P}$ of 300 Hz and an exposure time of 0.5 ms. This results in a longitudinal data point distance $dx_{P}$ of 6.981 mm. Each laser triangulation sensor measures distance and intensity with a sampling frequency $f_{S}$ of 20 kHz. With a ring speed of $20\;\min^{-1}$, the longitudinal data point distance $dx_{S}$ becomes 0.105 mm. The fixed sensors measure on lines with a lateral distance of 10 mm to each other. The movable sensor can be positioned laterally over the entire ring width according to a command signal $y_{S}$ for the lateral position.

A lateral disturbance movement is introduced using the lateral deflection system. The system is deflected by amplitude $a_{L}$ of ± 10.5 mm, starting from the middle of the ring. A lateral disturbance movement is applied by a triangle wave movement with lateral wavelengths $\lambda_{L}$ in the range between ~20 m and ~100 m. The frequency $f_{L}$ of the lateral deflection is obtained using the ring velocity $v_{R}$:

(1) $$f_{L}=\frac{v_{R}}{\lambda_{L}}$$

The parameters of the lateral disturbance movement applied by the lateral deflection system are indicated in Table 2.

**Figure 8:**
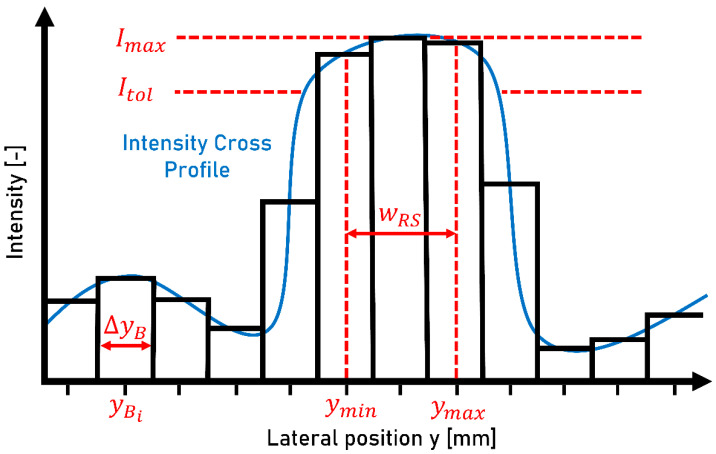
The schematic intensity cross-profile of the laser profilometer and the discretized intensity cross-profile represented as blocks. $\Delta y_{B}$: block width of the discretized intensity cross-profile; $y_{B_{i}}$: lateral position of the i-th block, $I_{max}$: maximum intensity of the cross-profile; $I_{tol}$: threshold intensity for the determination of the running surface; $y_{min}$: left boundary of the running surface; $y_{max}$: right boundary of the running surface; $w_{RS}$: width of the running surface.

A target position $y_{s}$ for the moving sensor is calculated from the measured transverse intensity profile (transverse to the direction of travel) of the laser profilometer. The bright, reflective, and dry surface of the running surface causes a visible intensity maximum. An illustrative example of an intensity profile is shown in Figure 8.

To provide the most stable running surface detection possible, the intensity profile is pre-filtered using a median filter with a rank of 100. To prevent individual peaks from influencing the target position $y_{S}$ excessively, the intensity profile is divided into blocks. Each block has a width $\Delta y_{B}$ of 2 mm and is assigned a central position $y_{B_{i}}$. The mean intensity is determined for each block. The maximum of these block intensities $I_{max}$ is evaluated based on all blocks. To define the width of the running surface, the block with maximum intensity cannot only be considered. A range must be defined, in which blocks are assigned to the running surface. Starting from the maximum intensity $I_{max}$, the threshold value $I_{tol}$ was calculated with a tolerance t of 5%:

(2) $$I_{tol}=I_{max}\cdot \left(1-t\right)=0.95\cdot I_{max}$$

If the condition is fulfilled that a block intensity $I_{i}$ is within the range $I_{tol}<I_{i}<I_{max}$ and the blocks are adjacent to each other, they are assigned to the running surface. N represents the total number of blocks within the running surface. The minimum value for $y_{B_{i}}$ from N blocks corresponds to the boundary of the running surface $y_{min}$. The boundary $y_{max}$ results from $y_{min}$ and the number of blocks N:

(3) $$y_{max}=y_{min}+N\cdot \Delta y_{B}$$

The values of the boundaries $y_{min}$ and $y_{max}$ of the running surface determine the running surface width $w_{RS}$:

(4) $$w_{RS}=y_{max}-y_{min}$$

The command signal $y_{0}$ corresponding to the centerline of the lateral position is calculated as:

(5) $$y_{0}=y_{min}+\frac{w_{RS}}{2}$$

The target position $y_{C}$ of the controller differs slightly from the command signal $y_{0}$ since the maximum step size of the moving sensor is limited to 8 mm.

## 3. Results

### 3.1. Surface Condition Detection

**Figure 9:**
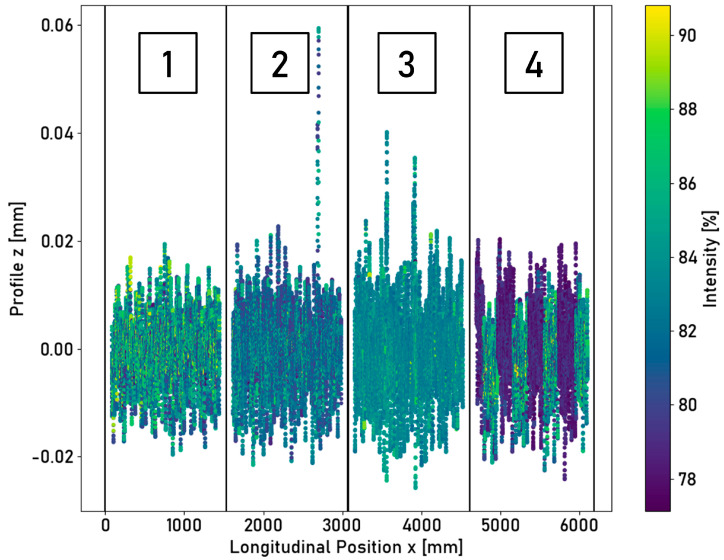
The longitudinal profile of the ring surface measured using a single laser triangulation sensor. The individual data points are colored according to their measured intensity. The ring is divided into four segments, each with a unique corrosion pattern. 1—metallic, 2—corroded, 3—longitudinal split, 4—alternation.

A single laser triangulation sensor was applied to detect different surface conditions. No distinctive differences between various surface conditions were obtained from the measured longitudinal profile. Differences can be found for the simultaneously measured intensity of the light spot reflected to the receptor element. The measured longitudinal profile and corresponding intensities are shown in Figure 9.

Differences between different surface states are visually recognizable for alternation between corroded and non-corroded surfaces within the fourth segment of the ring. For further segments, a difference is more difficult to identify visually, and the statistical characteristics of the measured intensity are to be considered. The statistical parameters of the measured intensity for the different ring segments are summarized in Table 3.

**Figure 10:**
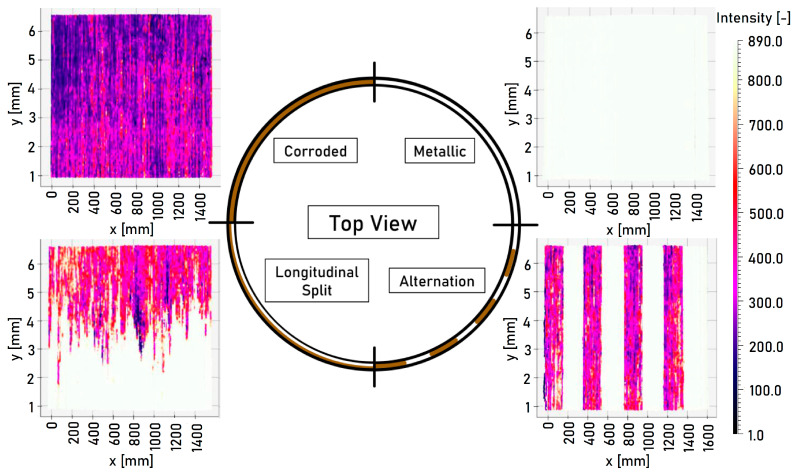
Intensity measurement of the ring taken using the laser profilometer. The ring is divided into four segments, each with a unique corrosion pattern. 1—metallic, 2—corroded, 3—longitudinal split, 4—alternation.

In the following, the intensity is measured with the laser profilometer along a line transverse to the direction of travel. The measured intensities for the different segments of the ring and the varying surface conditions are shown in Figure 10.

The measured intensity is represented in RGB color space. A clear qualitative distinction can be made between corroded and non-corroded surfaces. The longitudinally split segment is correctly resolved, and optical differentiation in the lateral direction is possible. Narrow alternations between corroded and non-corroded surfaces are distinguishable (alternation segment).

### 3.2. Running Surface Detection

**Figure 11:**
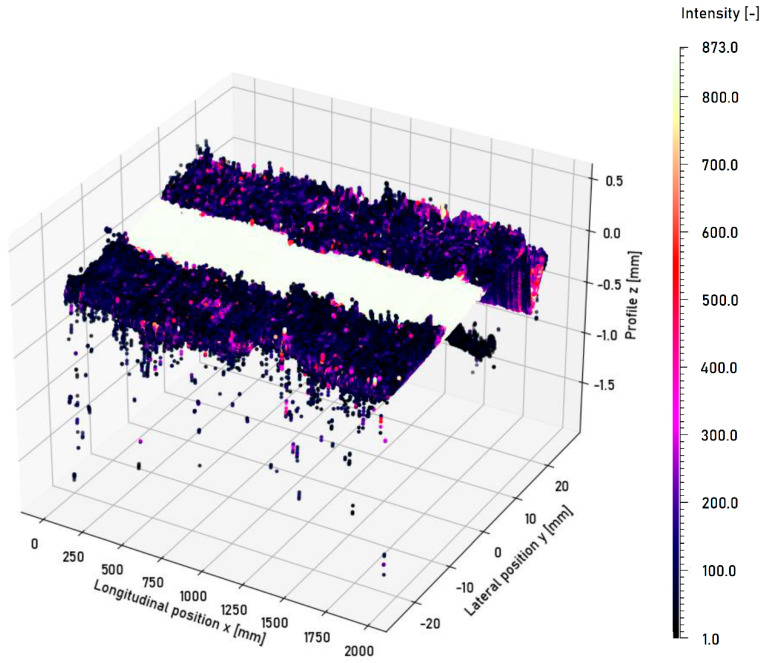
Surface profile measurement of the ring surface with an artificial running surface carried out using the laser profilometer. The individual data points are colored according to the measured intensity.

In the subsequent section, the surface with an artificial running surface shown in Figure 7 is considered. The artificial running surface is qualitatively distinguishable from the corroded edge areas using the intensity measurements of the laser profilometer. The measured intensities are shown in Figure 11.

**Figure 12:**
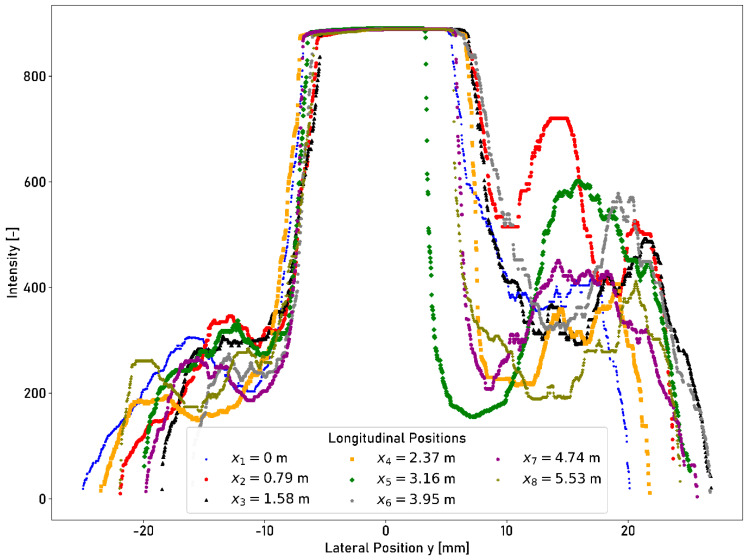
Cross-profile intensity measurements taken using the laser profilometer at eight different longitudinal positions of the ring.

The quantitative analysis of the intensities is performed by evaluating eight profile cross-sections in the circumferential direction of the ring. The intensity profile transverse to the direction of travel for eight different positions along the ring is shown in Figure 12.

The intensity is expressed numerically in values between 0 (minimum intensity) and 1023 (maximum intensity). A stable plateau of maximum intensity can be determined, indicating the running surface as it is shown in Figure 12. The intensity of the plateau assumes a value of 889 for all eight measured cross-sections at different longitudinal positions $x_{1\ldots 8}$. The left boundary of the running surface $y_{min}$ varies between −7.094 mm at longitudinal position $x_{4}$ and −5.404 mm at longitudinal position $x_{3}$. The right boundary of the running surface $y_{max}$ varies between 3.292 mm at longitudinal position $x_{5}$ and 6.987 mm at longitudinal position $x_{2}$. The measured intensities show another local maximum in the corroded edge area starting from the lateral position of approx. 10 mm. The highest peak assumes a value of 720 at a lateral position of 19.116 mm and longitudinal position $x_{4}$. The lowest peak assumes a value of 407 at a lateral position of 14.283 mm and longitudinal position $x_{2}$. The peaks beyond the right boundary of the running surface are caused when there is less coverage of the ground surface of the corroded layers. If the running surface width is determined from the transverse profile of the intensity according to the process described in Section 2.4, the average width of the running surface is 12.22 mm. The standard deviation measures 1.10 mm.

**Figure 13:**
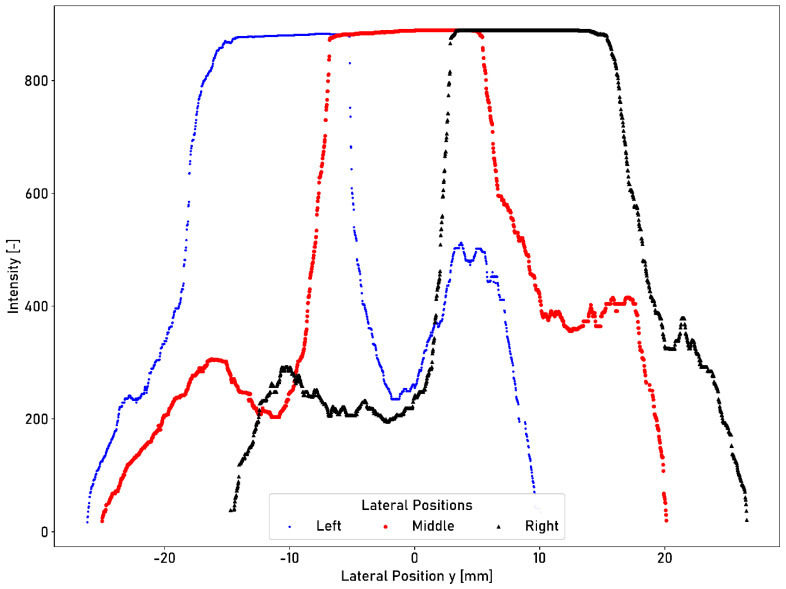
Cross-profile intensity measurements taken using the laser profilometer at three different lateral positions (±10.5 mm and central position) at the same longitudinal position of the ring.

The intensity plateau must be visible if the laser profilometer is not positioned centrally above the running surface. Intensity profiles can be measured at the identical longitudinal position with different lateral deflections of −10.5 mm (left position), 0 mm (central position), and +10.5 mm (right position). The measured intensity profiles for different lateral deflections are shown in Figure 13.

The intensity plateau of the running surface is preserved at the left and right lateral positions compared to the central position. In the deflected positions, the plateau seems slightly flattened at the edges, as shown in Figure 13. The number of acquired data points is reduced for the deflected positions. At the central position, 1696 data points are acquired. If the position is shifted to the left, 1362 data points are acquired; moreover, if the position is shifted to the right, 1555 data points are acquired. The measured intensities show a local peak beyond the right boundary of the running surface, as shown in Figure 12. The measurement recorded at a deflection to the right attenuates the peak. A peak value of 415 is acquired in the central position. The peak value increases to 512 for a deflection to the left position. Altered reflection conditions due to the deflection cause the amplification or attenuation of peaks.

**Figure 14:**
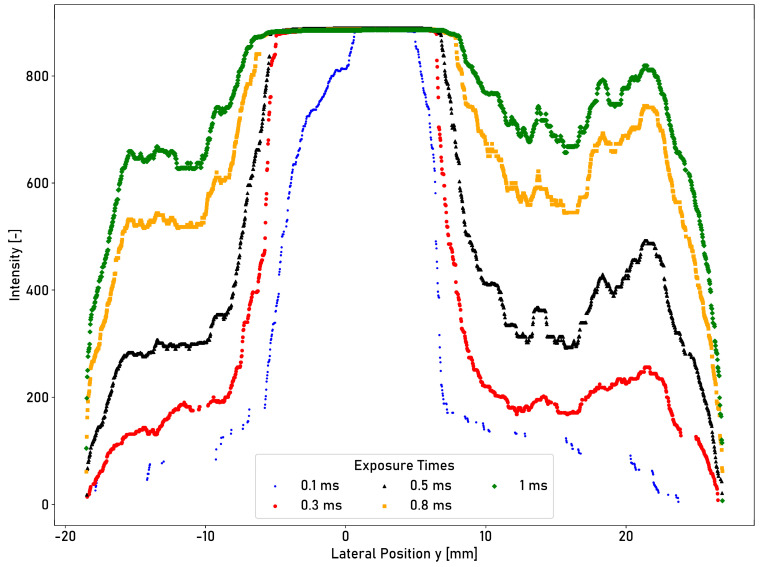
Cross-profile intensity measurements taken using the laser profilometer at the identical longitudinal position of the ring and different exposure times.

Different exposure times influence the measured cross-profile of the intensity of the artificial running surface. Figure 14 shows the measured cross-profile of the intensity at the same longitudinal position with exposure times 0.1 ms, 0.3 ms, 0.5 ms, 0.8 ms, and 1 ms.

The plateau of the intensity of the artificial running surface (compared to measurements with 0.5 ms) is not preserved for an exposure time of 0.1 ms. The running surface width is determined from the transverse profile of the intensity according to the process described in Section 2.4. The determined running surface width increases from 4.18 mm at 0.1 ms to 12.35 mm at 0.5 ms and 15.53 mm at 1 ms. For exposure times of 0.8 ms and 1 ms, the differences in intensity levels between the running surface and the corroded edge areas are smaller. The mean intensity values over the entire profile range increase from 514.72 at 0.5 ms to 673.22 at 0.8 ms and 744.66 at 1 ms. The number of measured data points decreases with decreasing exposure time. The intensity cross-profile for an exposure time of 0.1 ms shows visible gaps, as shown in Figure 14. For an exposure time of 0.1 ms, 603 data points are acquired; however, for an exposure time of 1 ms, 1751 data points are acquired.

### 3.3. Lateral Compensation

The measured tactile roughness $R_{a}$ close to the centerline of the ring is 3.68 μm. For the edge areas, the roughness $R_{a}$ without corrosion is 2.80 μm for the area towards the outside of the ring and 2.88 μm towards the inside of the ring. The width of the running surface $w_{RS}$ is determined based on the procedure described in Section 2.4. The determined widths that depend on the different lateral deflection movements are stated in Table 4.

**Figure 15:**
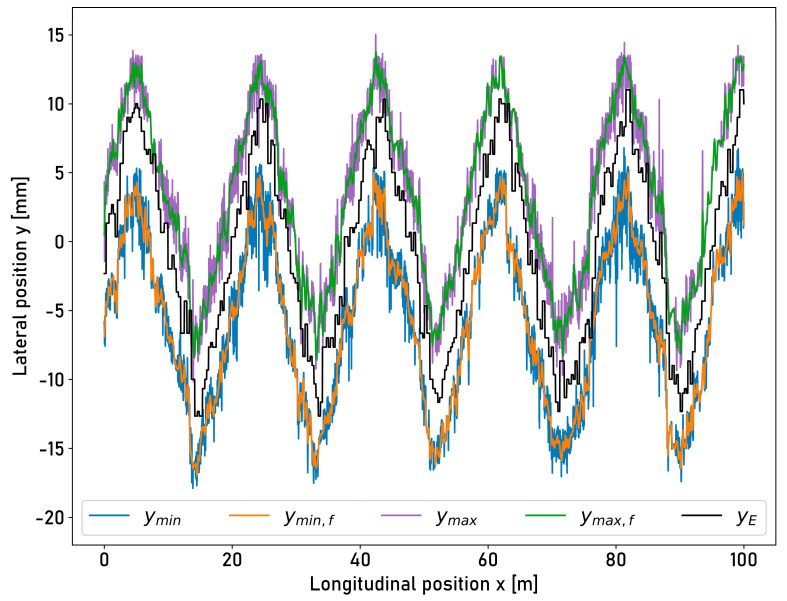
Illustration of determined running surface boundaries from laser profilometer intensities and the actual lateral position of the laser displacement sensor while the compensation setup is moving laterally with an amplitude of 10.5 mm and a wavelength of 18.85 m for a length of 100 m. $y_{min}$: left running surface boundary;$\;y_{min,f}$: left running surface boundary (filtered); $y_{max}$: right running surface boundary; $y_{max,f}$: right running surface boundary (filtered); $y_{E}$: actual lateral position of the measuring laser displacement sensor (encoder).

The mean width of the running surface across all programs is 9.19 mm with a mean standard deviation of 1.55 mm. The lateral position of the centerline is calculated based on the positions of the edges $y_{min}$ and $y_{max}$ of the running surface. The centerline is used to control the position of the moving sensor $y_{E}$. Figure 15 shows the measurement at a length of 100 m and at the shortest wavelength of the lateral triangle wave deflection motion with a wavelength $\lambda_{L}$ of 18.85 m.

**Figure A2:**
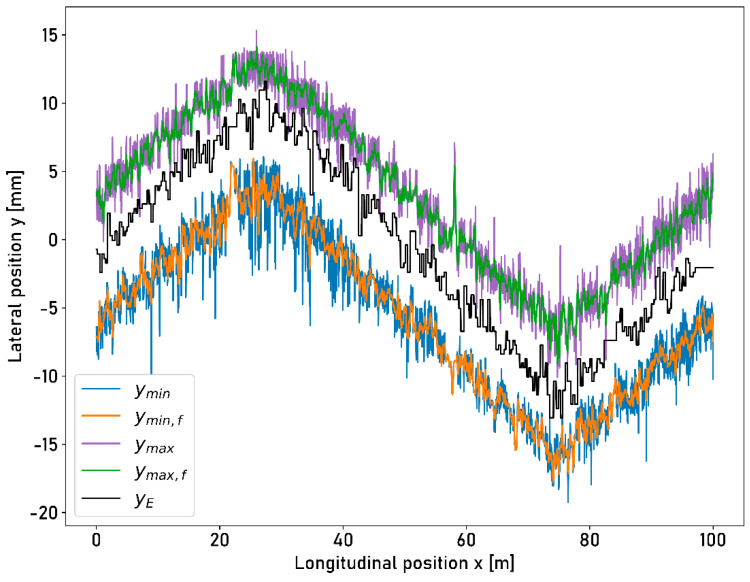
Illustration of determined running surface boundaries from laser profilometer intensities and the actual lateral position of the laser displacement sensor while the compensation setup is moving laterally with an amplitude of 10.5 mm and a wavelength of 97.70 m for a length of 100 m. $y_{min}$: left running surface boundary;$\;y_{min,f}$: left running surface boundary (filtered); $y_{max}$: right running surface boundary; $y_{max,f}$: right running surface boundary (filtered); $y_{E}$: actual lateral position of the measuring laser displacement sensor (encoder).

**Figure A3:**
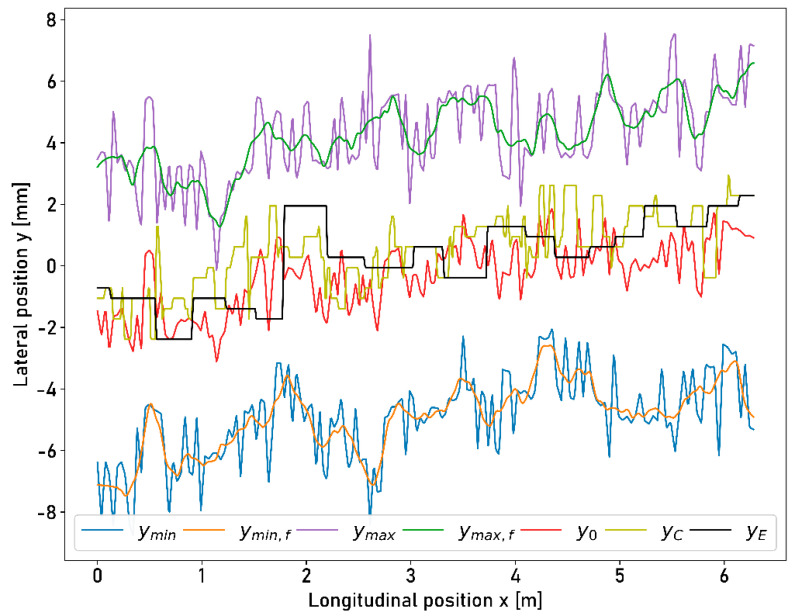
Illustration of the determined running surface boundaries from laser profilometer intensities, the theoretical set point, the practical set point, and the actual lateral position of the laser displacement sensor while the compensation setup is moving laterally with an amplitude of 10.5 mm and a wavelength of 97.70 m for one ring rotation. $y_{min}$: left running surface boundary; $y_{min,f}$: left running surface boundary (filtered); $y_{max}$: right running surface boundary; $y_{max,f}$: right running surface boundary (filtered); $y_{0}$: theoretical set point from profilometer intensities, $y_{C}$: actual set point, $y_{E}$: actual lateral position of the measuring laser displacement sensor (encoder).

The positions of the edges of the running surface filtered using a Savgol filter are shown additionally as $y_{min,\;f}$ and $y_{max,f}$. The measurements that were 100 m in length at longer wavelengths are shown in Figure A2, Figure A3, Figure A5, and Figure A7 in Appendix B. Visually, a phase shift between the edges of the running surface and the position of the moving sensor $y_{E}$ is identifiable, where the sensor is mostly positioned inside the running surface. Table 5 shows the percentages of all data points that are outside of the running surface or exceed an edge position.

**Figure 16:**
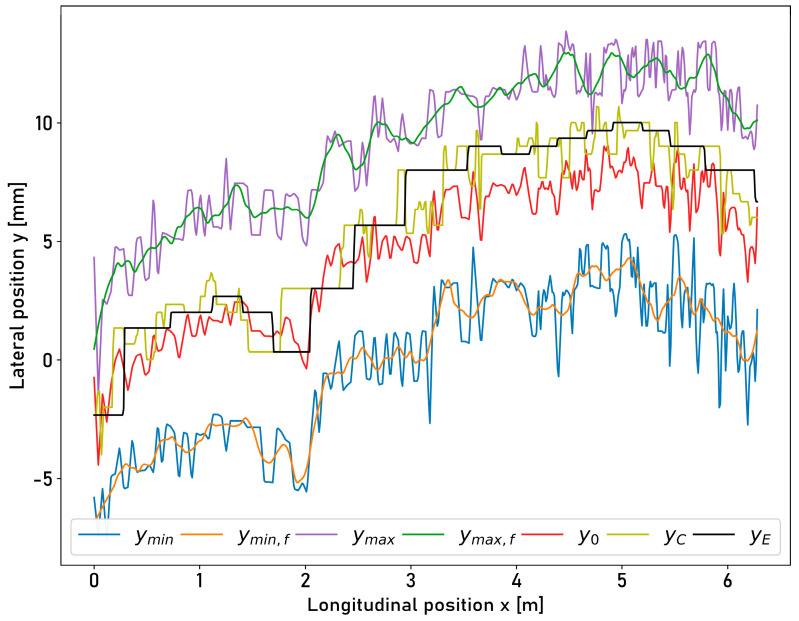
Illustration of the determined running surface boundaries from laser profilometer intensities, the theoretical set point, the practical set point, and the actual lateral position of the laser displacement sensor, while the compensation setup is moving laterally with an amplitude of 10.5 mm and a wavelength of 18.85 m for one ring rotation. $y_{min}$: left running surface boundary; $y_{min,f}$: left running surface boundary (filtered); $y_{max}$: right running surface boundary; $y_{max,f}$: right running surface boundary (filtered); $y_{0}$: theoretical set point from profilometer intensities; $y_{C}$: actual set point; $y_{E}$: actual lateral position of the measuring laser displacement sensor (encoder).

Without lateral deflections, 0.04% of the data points are positioned outside the running surface. For lateral disturbance movements in the wavelength range of 78.07 m and 97.70 m, 0.40% and 0.51% of the data points are positioned outside the running surface. For the shortest wavelength of 18.85 m, an increase to 1.56% can be observed. The boundary $y_{min}$ is undershot between 0.00% and 0.36% of the data points. The boundary $y_{max}$ is exceeded between 0.02% and 1.20% of the data points. Figure 16 shows a detailed view for one ring rotation of the measured lateral positions of the running surface edges and the lateral position of the moving sensor at the shortest wavelength of the lateral triangle wave deflection motion, with a wavelength $\lambda_{L}$ of 18.85 m.

**Figure A4:**
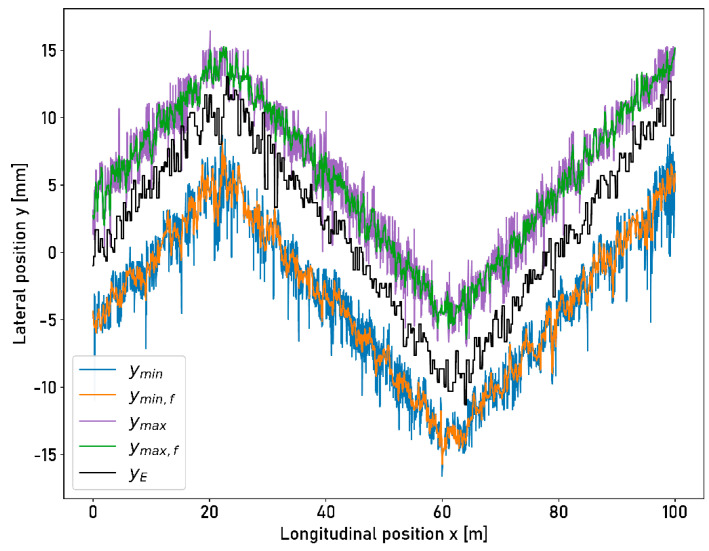
Illustration of determined running surface boundaries from laser profilometer intensities and the actual lateral position of the laser displacement sensor while the compensation setup is moving laterally with an amplitude of 10.5 mm and a wavelength of 78.07 m for a length of 100 m. $y_{min}$: left running surface boundary;$\;y_{min,f}$: left running surface boundary (filtered); $y_{max}$: right running surface boundary; $y_{max,f}$: right running surface boundary (filtered); $y_{E}$: actual lateral position of the measuring laser displacement sensor (encoder).

**Figure A5:**
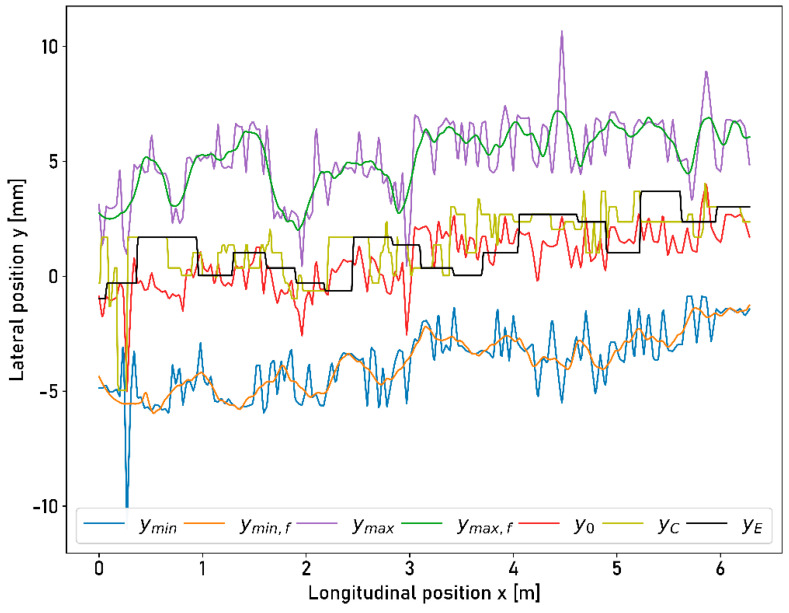
Illustration of the determined running surface boundaries from laser profilometer intensities, the theoretical set point, the practical set point, and the actual lateral position of the laser displacement sensor while the compensation setup is moving laterally with an amplitude of 10.5 mm and a wavelength of 78.07 m for one ring rotation. $y_{min}$: left running surface boundary; $y_{min,f}$: left running surface boundary (filtered); $y_{max}$: right running surface boundary; $y_{max,f}$: right running surface boundary (filtered); $y_{0}$: theoretical set point from profilometer intensities, $y_{C}$: actual set point, $y_{E}$: actual lateral position of the measuring laser displacement sensor (encoder).

**Figure A6:**
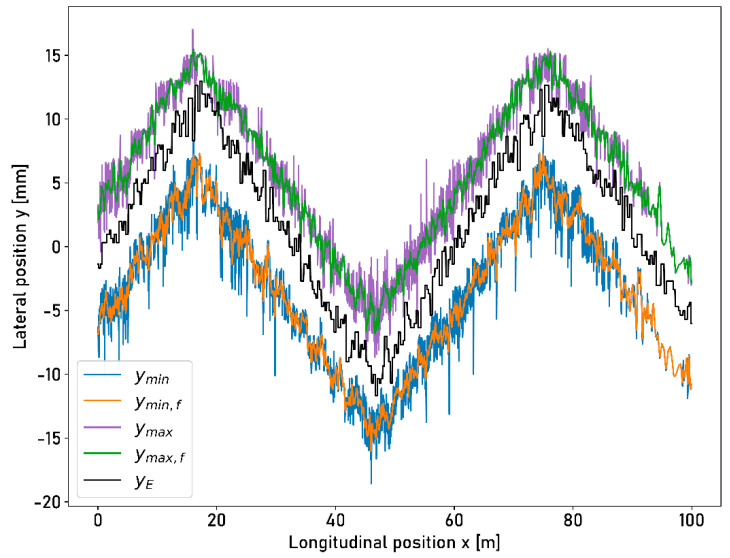
Illustration of determined running surface boundaries from laser profilometer intensities and the actual lateral position of the laser displacement sensor while the compensation setup is moving laterally with an amplitude of 10.5 mm and a wavelength of 58.74 m for a length of 100 m. $y_{min}$: left running surface boundary;$\;y_{min,f}$: left running surface boundary (filtered); $y_{max}$: right running surface boundary; $y_{max,f}$: right running surface boundary (filtered); $y_{E}$: actual lateral position of the measuring laser displacement sensor (encoder).

**Figure A7:**
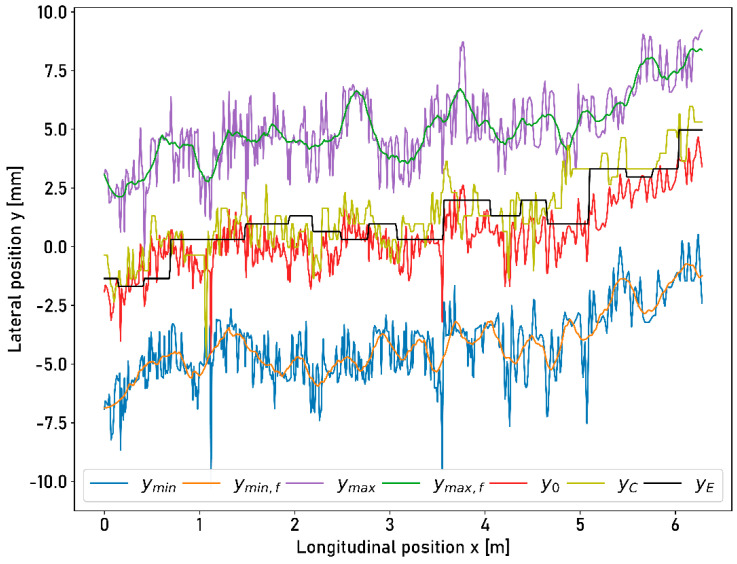
Illustration of the determined running surface boundaries from laser profilometer intensities, the theoretical set point, the practical set point, and the actual lateral position of the laser displacement sensor while the compensation setup is moving laterally with an amplitude of 10.5 mm and a wavelength of 58.74 m for one ring rotation. $y_{min}$: left running surface boundary; $y_{min,f}$: left running surface boundary (filtered); $y_{max}$: right running surface boundary; $y_{max,f}$: right running surface boundary (filtered); $y_{0}$: theoretical set point from profilometer intensities, $y_{C}$: actual set point, $y_{E}$: actual lateral position of the measuring laser displacement sensor (encoder).

**Figure A8:**
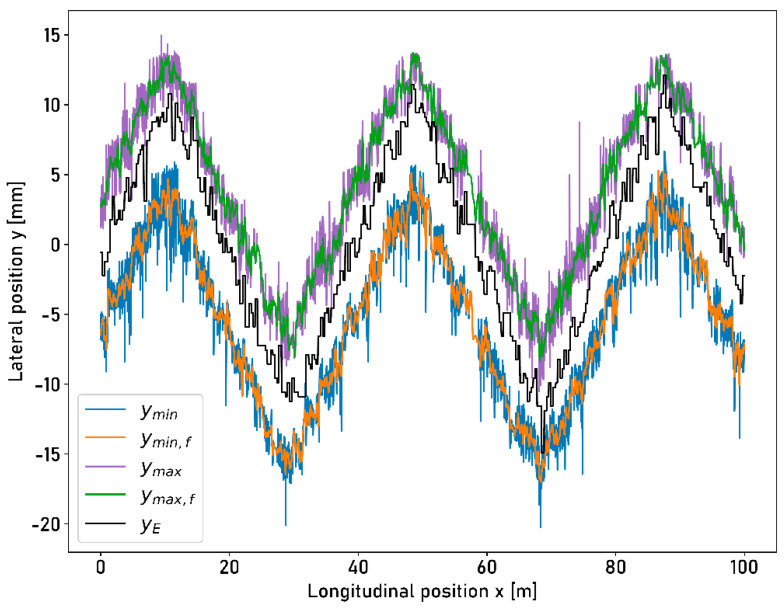
Illustration of determined running surface boundaries from laser profilometer intensities and the actual lateral position of the laser displacement sensor while the compensation setup is moving laterally with an amplitude of 10.5 mm and a wavelength of 38.93 m for a length of 100 m. $y_{min}$: left running surface boundary;$\;y_{min,f}$: left running surface boundary (filtered); $y_{max}$: right running surface boundary; $y_{max,f}$: right running surface boundary (filtered); $y_{E}$: actual lateral position of the measuring laser displacement sensor (encoder).

Additionally, the centerline $y_{0}$ of the running surface and the controller target position $y_{C}$ are shown. The measurements for one ring rotation at longer wavelengths are shown in Figure A2, Figure A4, Figure A6, and Figure A8 in Appendix B. Deviations can be seen visually between the actual position $y_{E}$ and the targeted centerline $y_{0}$. The deviations of the actual position of the moving sensor $y_{E}$ from the position of the theoretical centerline $y_{0}$ of the running surface and the corresponding standard deviation are provided in Table 6.

If the average deviation is averaged over all lateral deflections, the average positioning deviation is 1.24 mm.

## 4. Discussion

### 4.1. Surface Condition Detection and Running Surface Detection

The individual laser triangulation sensors are only suitable to a limited extent for distinguishing between corroded and non-corroded surfaces. No distinction can be derived solely from distance measurements. The intensity measurements allow for a distinction to be made via the statistical characteristic values of a defined data segment. The standard deviation of the intensity for a metallic surface has a value of 3.21%, while this value drops to 1.00% for a corroded surface. The laser power is quickly regulated to a target value by the device. The segment of alternation implies rapid changes in the surface condition, which can be detected. A distinction can already be visually derived from Figure 9 for the alternation segment. The standard deviation has a value of 3.36%. The laser triangulation sensor is only suitable to a limited extent for detecting the running surface or for the detection of fast-changing surface conditions. Since only the intensity at a discrete lateral position can be measured, no statement about the exact position of the running surface or the width of the running surface can be determined.

The laser profilometer offers the advantage that an entire laser line can be projected onto the surface transverse to the direction of travel; thus, the intensity can be measured on the entire width of the rail head simultaneously. The qualitative analysis shows that the different surface conditions can be detected using the intensity measurements obtained using the laser profilometer. An identifiable high intensity plateau (value of approx. 889 on a scale from 0 to 1023) is evident, representing the artificial running surface. Lateral displacements of the laser profilometer influence the intensity profile. A flattening of the profile occurs, as well as the attenuation and amplification of peaks in the corroded edge areas. A local intensity peak is measured beyond the right boundary of the running surface due to the irregular thickness of the corroded edge layers. A value of 415 is measured for the central position. The measured intensity increases by 97 for a deflection to the left position. The discrepancy is caused by varying reflection conditions, which are imposed by lateral deflection and correlate with the number of acquired data points. For the central position, 1696 data points are acquired; however, for the left position, it is reduced by 334 data points. The exposure time must be set manually and constantly for the measurement. This parameter has a significant influence on the result. The entire intensity plateau is no longer detectable at an exposure time of 0.1 ms. Not enough light is reflected to the receptor element. The number of acquired data points at an exposure time of 0.1 ms is reduced by approx. 65.6% compared to a measurement at an exposure time of 1 ms. If the exposure time is increased, the differences between the running surface and the edge areas increasingly disappear. This can be observed in the average of the intensity profile. The average intensity is 514.72 at an exposure time of 0.5 ms. It increases to 744.66 at an exposure time of 1 ms. This corresponds to an increase of 44.7%. The detected width of the running surface increases with exposure time. At an exposure time of 1 ms, the width increases by 25.8% compared to an exposure time of 0.5 ms. It is possible to calculate not only the position of the running surface but also its width $w_{\mathrm{RS}}$. If the width of the running surface is calculated based on the method proposed in Section 2.4 and eight measurements while the ring is not rotating, the width of the running surface becomes, approximately, 12.22 mm. If the width of the running surface is determined automatically based on a transverse profile subdivided into blocks to identify an intensity plateau as stable as possible, an average value of 9.31 mm is obtained without a lateral disturbance movement. The different values arise due to the subdivision of the profile into blocks with a width of 2 mm. Blocks are only assigned to the running surface if their average intensity is within the defined range $I_{\mathrm{tol}}<I_{i}<I_{\max}$. The blocks at the boundaries possess a lower average intensity $I_{i}$ and are, consequently, not assigned to the running surface. The automatic methodology, therefore, determines a more conservative estimate of the running surface width. Lateral disturbances have no relevant influence on the determined width of the running surface. The determined widths are in the range between 9.14 mm and 9.25 mm, with standard deviations between 1.51 mm and 1.54 mm.

The width of the projected line is crucial for the quality of the running surface detection, especially considering lateral deflections. If the setup is deflected by 10.5 mm to the left or right, the intensity plateau flattens slightly at the edges. If the lateral deflection is increased further, the plateau collapses entirely. Parts of the emitted light are then no longer detected on the receptor element and are reflected into the surrounding area. When installing the profilometer on the train, care must be taken that it projects a line onto the rail head that is wide enough to measure at larger lateral deflections. A projected line width of at least 100 mm could be required for a UIC 60 rail head with a width of 72 mm. This problem is overlapped by vertical disturbance movements caused by the suspension of the train (primary suspension when installed on the bogie frame). The width of the projected line changes with the measuring distance. Thus, the line width is 44.3 mm at a measuring distance of 105 mm. At the reference distance of 125 mm, the line width is 51 mm. At a measuring distance of 145 mm, the width of the projected line is 57.8 mm. The reference distance of 125 mm can only be kept constant for laboratory tests. The influence of the curvature of the rail head surface on the intensity measurement has to be verified by tests on the train.

### 4.2. Lateral Compensation

Only 0.04% of the measured data points are outside the running surface for measurements without lateral disturbances. Lateral disturbances with wavelengths between 38.93 m and 97.70 m increase the error rate to approx. 0.50%. For a smaller wavelength of 18.85 m, 1.56% of the measured data points are positioned outside the running surface. A phase shift of the moving sensor compared to the controller target signal is visible in Figure 15 and Figure 16. It can be assumed that the technical limits of the installed components and the implemented controller are being reached at this point. For example, the maximum step size of the controller signal is limited. Consequently, the positioning error with respect to the centerline of the running surface shows an increase for lateral disturbance motions with shorter wavelengths. The positioning error increases from 1.21 mm at a wavelength of 97.70 m to 1.40 mm at a wavelength of 18.85 m. The positioning accuracy is affected by the selection of the block size $\Delta y_{B}$. The target signal is derived from the central position of the respective block. The smaller the block length selected, the more precise the positioning can be. On the other hand, the positioning becomes increasingly unstable since individual outliers in the profile influence the determined maximum value and thus the target signal.

It must be considered that, during the measurement on the train, additional external disturbances such as humidity and vibrations are to be expected. The actuator system would have to be designed to be sufficiently robust and stiff to avoid the disturbance of the measurement results. The laser profilometer must be positioned in front of the measurement setup in the direction of travel to achieve minimum delay time between running surface detection and the positioning of the sensors above the running surface. It is recommended to install two laser profilometers to cover both driving directions. In the laboratory test series, a visible running surface with already clearly corroded edge areas is assumed. In the rail network, freshly ground or recently ground rail heads can be encountered. Additionally, the running surface may be subject to impurities, small accumulations of corrosion, or indentations in the rail network, which may additionally influence the measurement. In this case, it is useful to include the simultaneously measured transverse profile of the rail head for positioning.

The maximum possible sampling frequency of the profilometer is not only limited by the measuring range to be achieved but also by the performance of the setup. The current laboratory setup can achieve a maximum sampling frequency of 300 Hz because of the amount of data to be processed. At a speed of $7.5{\;\mathrm{km}\;h}^{-1}$, this corresponds to a longitudinal distance of 6.98 mm between the measured intensity profiles.

## 5. Conclusions

Measurements of the acoustic rail roughness must be performed within the running surface despite the lateral disturbing movements of the train. Various concepts have been presented based on combinations of the available sensors to acquire the width and the lateral position of the running surface. A single laser triangulation sensor is only suitable to a limited extent for distinguishing different surface states based on the measured intensity. For a metallic surface, the standard deviation of the intensity was 3.21%, whereas the value for the corroded surface was 1.00%. A laser profilometer could clearly distinguish between the corroded surfaces of the edge areas and the non-corroded surface, called the running surface, based on the measured intensity. A stable plateau with an intensity of 889 (on a scale between 0 and 1023) was measured for the running surface. For the corroded edge areas, intensities considerably below the plateau of 889 were measured and varied in their quality of coverage of the corrosion layer. Consequently, the concept of a moving measurement line consisting of four laser triangulation sensors and a laser profilometer was evaluated experimentally. Both the width and the lateral position of the running surface could be determined. The setup achieved a maximum mean positioning error of 1.40 mm with respect to the target signal (centerline of the running surface) at a driving speed of $7.5{\;\mathrm{km}\;h}^{-1}$ and a lateral disturbance movement with a wavelength of 18.85 m. Moreover, 98.44% of the measured data points are located inside the running surface.

The system must be validated on the train at line speed, considering the external disturbances that occur. Various forms of the running surface must be considered. In the future, the installation of the proposed running surface detection concept could provide information about different positions of the running surface on the rail head. Relations between operational parameters such as vehicle types, etc., and the position of the running surface could be systematically investigated. The detection of the lateral position of the running surface enables the application of an optical measuring system for the systematic detection of the rail roughness and thus an improvement in the rail network’s condition.

## Figures and Tables

**Table 1:** Overview of the measuring distances and measuring ranges of the Micro-Epsilon sensors installed. $d_{S}$: measurement distance of the sensor (mid-range); $a_{S}$: measuring range of the sensor.

Sensor	$d_{S}\;\left[\mathrm{mm}\right]$	$a_{S}\;\left[\mathrm{mm}\right]$
scanCONTROL 3060–50/BL	125	40
optoNCDT 2300–10 LL	35	10

**Table 2:** The parameters of the triangle wave movement, with which the lateral movement of the train is simulated. $f_{L}$: frequency of the lateral disturbance movement; $\lambda_{L}$: wavelength of the lateral disturbance movement; $a_{L}$: amplitude of lateral disturbance movement.

Experiment	$f_{L}\;\left[\mathrm{Hz}\right]$	$\lambda_{L}\;\left[m\right]$	$a_{L}\;\left[\mathrm{mm}\right]$
1	0.02	97.70	10.5
2	0.03	78.07	10.5
3	0.04	58.74	10.5
4	0.05	38.93	10.5
5	0.11	18.85	10.5

**Table 3:** Descriptive analysis of the intensity of the ring measured using a laser triangulation sensor. The ring is divided into four segments, each with a unique corrosion pattern.

Segment and Surface Condition	Mean [%]	Standard Deviation [%]	Minimum [%]	Maximum [%]
1—Metallic	83.74	3.21	76.74	90.91
2—Corroded	79.09	1.00	76.64	83.19
3—Longitudinally Split	78.14	0.65	74.58	82.40
4—Alternation	81.15	3.36	77.13	90.81

**Table 4:** The mean running surface width $w_{RS}$ and standard deviation for different lateral deflection movements determined from laser profilometer measurements.

Experiment	$f_{L}\;\left[\mathrm{Hz}\right]$	$\lambda_{L}\;\left[m\right]$	$\mathrm{Mean}\;w_{RS}$ [mm]	$\mathrm{Standard}\;\mathrm{Deviation}\;w_{RS}$ [mm]
Undisturbed	-	-	9.31	1.68
1	0.02	97.70	9.16	1.54
2	0.03	78.07	9.15	1.54
3	0.04	58.74	9.15	1.51
4	0.05	38.93	9.25	1.54
5	0.11	18.85	9.14	1.51

**Table 5:** The percentage of data points at which the position of the moving sensor $y_{E}$ is less than $y_{min}$ or greater than $y_{max}$.

Experiment	$f_{L}\;\left[\mathrm{Hz}\right]$	$\lambda_{L}\;\left[m\right]$	$y_{E}>y_{max}$ [%]	$y_{E}<y_{min}$ [%]
Undisturbed	-	-	0.02	0.02
1	0.02	97.70	0.43	0.08
2	0.03	78.07	0.40	0.00
3	0.04	58.74	0.50	0.00
4	0.05	38.93	0.35	0.10
5	0.11	18.85	1.20	0.36

**Table 6:** The average deviation of the actual position of the sensor $y_{E}$ and the position of the centerline $y_{0}$ in the middle of the running surface as a function of the different deflection programs.

Experiment	$f_{L}\;\left[\mathrm{Hz}\right]$	$\lambda_{L}\;\left[m\right]$	Mean $\left|y_{E}-y_{0}\right|$ [mm]	$\mathrm{Standard}\;\mathrm{Deviation}\;\left|y_{E}-y_{0}\right|$ [mm]
Undisturbed	-	-	0.87	0.69
1	0.02	97.70	1.21	0.90
2	0.03	78.07	1.28	0.93
3	0.04	58.74	1.31	0.97
4	0.05	38.93	1.39	0.97
5	0.11	18.85	1.40	1.09

**Figure A9:**
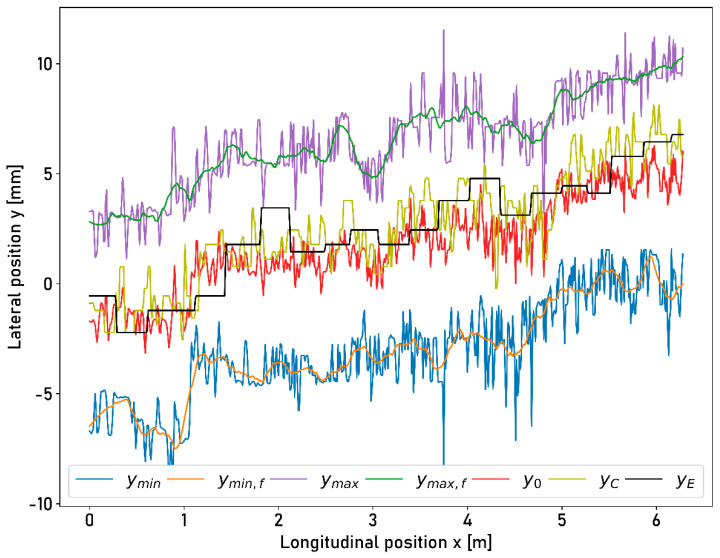
Illustration of the determined running surface boundaries from laser profilometer intensities, the theoretical set point, the practical set point, and the actual lateral position of the laser displacement sensor while the compensation setup is moving laterally with an amplitude of 10.5 mm and a wavelength of 38.93 m for one ring rotation. $y_{min}$: left running surface boundary; $y_{min,f}$: left running surface boundary (filtered); $y_{max}$: right running surface boundary; $y_{max,f}$: right running surface boundary (filtered); $y_{0}$: theoretical set point from profilometer intensities, $y_{C}$: actual set point, $y_{E}$: actual lateral position of the measuring laser displacement sensor (encoder).

## Data Availability

The data used in this study are available from the corresponding author upon request.

## Appendix A

This section contains supplementary figures to the main part. The experimental setup including the entire ring with artificial running surface is shown in Figure A1.

## Appendix B

This section contains supplementary illustrations to describe the edges of the running surface and the position of the moving sensor. In the following, various plots of measurements with a length of 100 m and one ring rotation, respectively, while the compensation setup is moving laterally with wavelengths 97.70 m, 78.07 m, 58.74 m, and 38.93 m, are shown. The lateral disturbance movements have an amplitude of 10.5 mm.
